# Harnessing pongamia shell hydrolysate for triacylglycerol agglomeration by novel oleaginous yeast *Rhodotorula pacifica* INDKK

**DOI:** 10.1186/s13068-020-01814-9

**Published:** 2020-10-19

**Authors:** Kukkala Kiran Kumar, Farha Deeba, Sauraj, Yuvraj Singh Negi, Naseem A. Gaur

**Affiliations:** 1 International Centre for Genetic Engineering and Biotechnology (ICGEB), New Delhi, 110067 India; 2 Department of Polymer & Process Engineering, Indian Institute of Technology Roorkee, Uttarakhand, 247667 India

**Keywords:** Pongamia shell hydrolysate, *Rhodotorula pacifica* INDKK, Microwave-aided Nile red screening, Triacylglycerol, Biodiesel

## Abstract

**Background:**

To meet the present transportation demands and solve food versus fuel issue, microbial lipid-derived biofuels are gaining attention worldwide. This study is focussed on high-throughput screening of oleaginous yeast by microwave-aided Nile red spectrofluorimetry and exploring pongamia shell hydrolysate (PSH) as a feedstock for lipid production using novel oleaginous yeast *Rhodotorula pacifica* INDKK.

**Results:**

A new oleaginous yeast *R. pacifica* INDKK was identified and selected for microbial lipid production. *R. pacifica* INDKK produced maximum 12.8 ± 0.66 g/L of dry cell weight and 6.78 ± 0.4 g/L of lipid titre after 120 h of growth, showed high tolerance to pre-treatment-derived inhibitors such as 5-hydroxymethyl furfural (5-HMF), (2 g/L), furfural (0.5 g/L) and acetic acid (0.5 g/L), and ability to assimilate C3, C5 and C6 sugars. Interestingly, *R. pacifica* INDKK showed higher lipid accumulation when grown in alkali-treated saccharified PSH (AS-PSH) (0.058 ± 0.006 g/L/h) as compared to acid-treated detoxified PSH (AD-PSH) (0.037 ± 0.006 g/L/h) and YNB medium (0.055 ± 0.003 g/L/h). The major fatty acid constituents are oleic, palmitic, linoleic and linolenic acids with an estimated cetane number (CN) of about 56.7, indicating the good quality of fuel.

**Conclusion:**

These results suggested that PSH and *R. pacifica* INDKK could be considered as potential feedstock for sustainable biodiesel production.

## Background

Global population is increasing exponentially and likely to reach ~ 8.6 billion by 2030, raising big concerns about energy [1]. Demand for plant oils has also been shooting up in parallel since they have many industrial applications like epoxy biopolymers [2], drug delivery systems [3], bio-lubricants [4], pharmaceuticals [4] and biodiesel [5]. However, plant oil-derived biodiesel production has raised many questions related to the sustainable use of food crops for cleaner energy production [6]. Therefore, oleaginous microbes having fatty acid profile similar to vegetable oils are considered as suitable alternative for biodiesel [7]. Among oleaginous microbes, micro-algae are being widely used for lipid production, but it requires vast region of land for large-scale cultivation, longer incubation time and specific light exposure [8]. Currently, oleaginous yeasts are of special interest as they can produce high lipid titres in short duration and require limited space [9]. Additionally, yeast has the ability to utilize various renewable carbon sources along with the potential to grow at low pH, which prevents bacterial contamination [10]. Together, these characteristics facilitate the development of oleaginous yeast-based technologies for economically attractive industrial process.

Presently *Rhodotorula*, *Rhodosporidium, Lipomyces* and *Trichosporon* are considered as potential yeast for lipid-based biodiesel production. Yeast strains belonging to these genera can accumulate intracellular lipids more than 60% of their dry cell weight (DCW), displayed tolerance to pre-treatment-derived inhibitors along with the ability to assimilate wide range of carbon sources [10]. Consequently, screening and identification of oleaginous yeast isolates from unexplored natural habitats are still relevant [10]. However, the cost of microbial oil production is exorbitant due to the high substrate cost [11]. Therefore, to establish a sustainable microbial lipid production, extra endeavours are requisite such that yeast efficiently utilize renewable and low-cost carbon sources [12]. In recent years, inexpensive lignocellulosic carbon sources like rice straw hydrolysate [13], elephant grass hydrolysate [14], sugarcane bagasse hydrolysate [15], groundnut shell hydrolysate [16], wheat straw [17] and waste office paper hydrolysates [18] have been used for microbial lipid production. But, the conversion of these renewable feedstock like lignocellulosic materials into lipids in a cost-effective manner is a key challenge [19]. The common steps involved in converting lignocellulosic materials to microbial lipids for biodiesel production include: hydrolysis of lignocellulosic materials into fermentable sugars; utilization of released sugars by microbes for lipid production; and biodiesel production from lipids. However, during thermo-chemical pre-treatment such as acid/alkali and steam explosion process, hydrolysates are laden with weak acids, furans and phenolic compounds like 5-hydroxymethylfurfural (5-HMF), furfural and acetic acid which inhibit yeast growth and lipid accumulation. Therefore, for removal of pre-treatment-generated inhibitors, detoxification methods like treatment with activated charcoal and over liming [20] are employed to facilitate efficient fermentation of hydrolysate sugars into lipids. Various studies focus on the utilization of the detoxified hydrolysate for the yeast lipid production. Patel et al. [21] explored lignocellulosic wastes such as *cassia fistula* for biodiesel production using *Rhodosporidium kratochvilovae*. Huang et al. [22] explored the rice straw hydrolysate which was detoxified by activated carbon for lipid production by *Trichosporon fermentans* with lipid titer of 12.1 g/L after 10 days fermentation. Recently, Liu et al. [17] reported lipid production on wheat hydrolysates using different oleaginous yeasts. Moreover, lignocellulosic hydrolysates contain wide range of C5 and C6 sugars including glucose, xylose and arabinose along with the inhibitors. Therefore, yeast isolates utilizing C5 and C6 sugars derived from low-cost feedstock along with the potential to tolerate high concentration of pre-treatment derived inhibitors are much essential for lipid production.

In this regard, *Pongamia pinnata* was explored as source of substrate for microbial lipid production. It is a non-edible oilseed tree, which belongs to Leguminosae family and grows in the semiarid regions such as Asia, Australia and Florida. It was estimated that one hectare of land can produce approximately 6.8 tons of seeds in shell which can generate 1.12 tons of oil, 1.9 tons of meal, 2.67 tons of pod shells and others [23]. The air-dried pongamia shells (PS) consist of 46.02% carbon, 0.23% nitrogen, 42.46% oxygen, 5.58% hydrogen and 5.7% ash [24]. However, the PS are generally discarded or burned after oil extraction from seeds. Recently, pongamia shells have been utilized as fuel briquettes [24], but their utilization as feedstock for biodiesel production remain unexplored. The compositional analysis of widely available waste PS unveils its potential application for lipid production by microbes.

Utilizing low-cost materials required development of more efficient process like employing trans-esterification process to produce a high-quality FAME biodiesel. FAME production process involves some advantages due to low energy utilization, flexibility in feedstock consumption, reduced capital cost and faster reaction by employing accelerated trans-esterification at lower temperature. However, renewable or green diesel produced via hydro-processing of vegetable oils involves costly additional steps of isomerization and cracking at higher temperature and pressure [25]. Interestingly, any alteration in the fatty acid profile influences the biodiesel properties during trans-esterification process. The relative fatty acids composition in oleaginous yeasts was found to be C18:1 > C16:0 > C18:2 = C18:0 that can be altered depending on the feedstock provided and their growth conditions [26]. The biodiesel quality is also affected by the refining process, production process and post-production parameters. Hence, international standards namely European (EN 14214), American Society for Testing and Materials (ASTM D6751) and Indian standards (IS15607-05) have been set up to monitor the parameters and quality of biodiesel. The important parameters for potential biodiesel are cetane number (CN), high heating value (HHV), cold filter plugging point (CFPP, °C), oxidative stability (OS, h), viscosity (mm^2^/s), iodine value (IV, mgI_2_/100 g), density (kg/m^3^) and saponification value (SV, mg KOH/g oil). The oil composition and biodiesel properties were evaluated in this study to ensure the biodiesel quality and compared to the ASTM, IS and EN biodiesel standards specifications.

The aim of this study was to explore an inexpensive and renewable raw material pongamia shell hydrolysate (PSH) for yeast lipid production. First, the high lipid-accumulating oleaginous yeast was screened using microwave-aided Nile red spectrofluorimetry method. Alkaline pre-treatment, acid pre-treatment, enzymatic hydrolysis, detoxification and lipid production by yeast fermentation in PSH were then conducted and optimized. To the best of our knowledge, this is the first report on lipid production from waste PSH using yeast fermentation. This study provides valuable information for researchers on microbial lipid production using PSH as sole carbon source.

## Results

### Screening and molecular identification of the selected yeast isolate

**Fig. 1:**
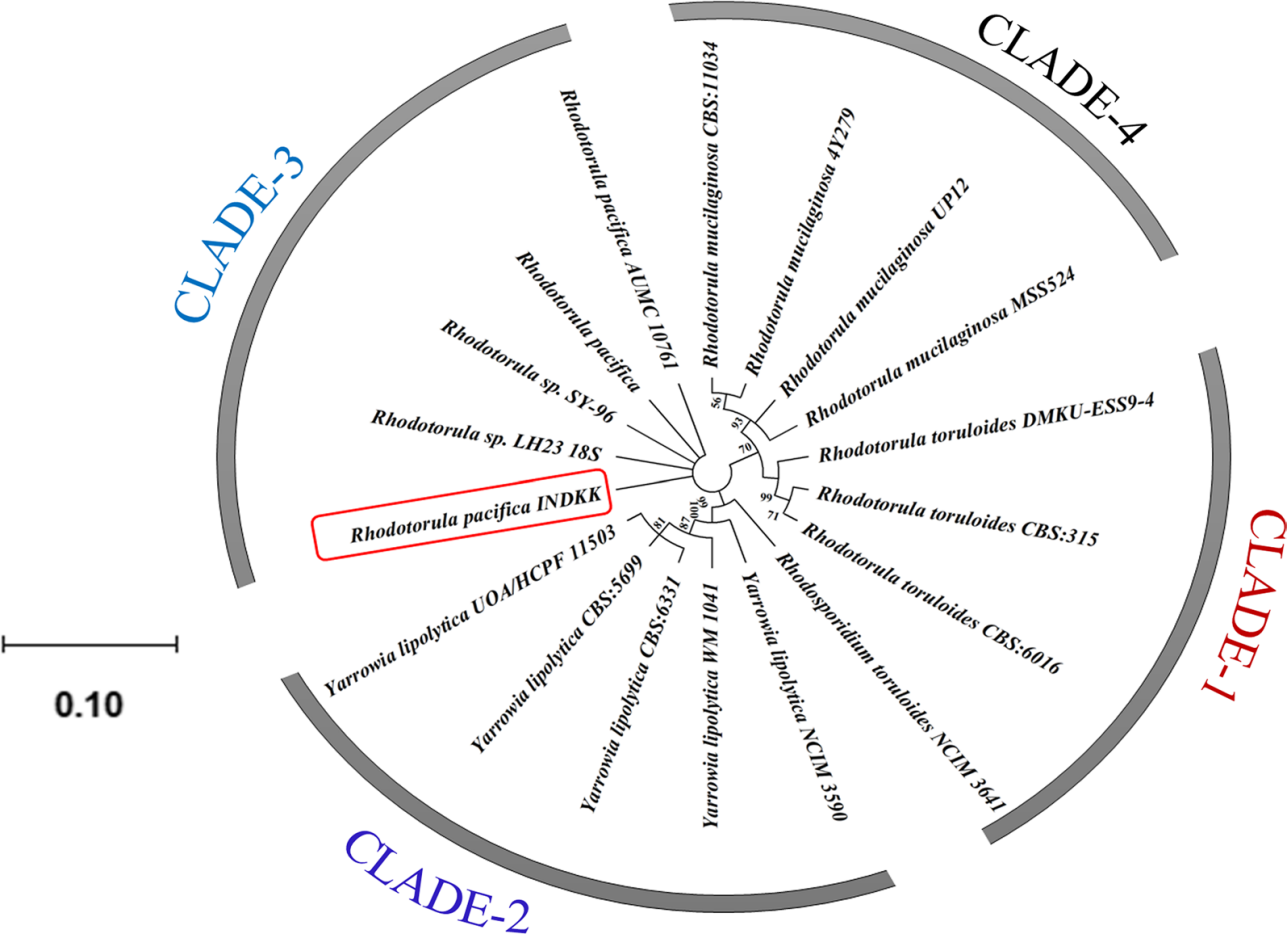
Phylogenetic tree of *Rhodotorula pacifica* INDKK (Acc. No. MN560184) showing evolutionary history constructed by MEGA X software using the maximum likelihood method. Bootstrap values greater than 50% are represented as numbers above the branches

In this study, we collected a pool of potential oleaginous yeast isolates (57) including strains procured from collection centres in India (NCIM and MTCC) as well as by screening samples from various sites of biomass degradation (Additional file 1: Table S1). Next, molecular identification of new yeast isolate was carried out by PCR amplification of the ITS region (using genomic DNA template) followed by phylogenetic relationship analysis. Top hits of the BLAST analysis showed 99% identity with yeast belonging to *Rhodotorula* species such as *Rhodotorula* sp. SY-96 (Acc. No. AB026006.2 of 1194 bp), *Rhodotorula* sp. LH23 (Acc. No. HQ832796.1 of 643 bp), *Rhodotorula pacifica* (Acc. No. AB193175.1 of 1194 bp) and *Rhodotorula pacifica* AUMC 10761 (Acc. no. KY495729.1 of 614 bp), indicating that selected isolate might belong to *Rhodotorula* species*.* Phylogenetic relationship with oleaginous yeasts was established by MEGA X software [27]. The evolutionary background of the taxa was determined by the maximum likelihood method [28]. The bootstrap consensus of tree was obtained from 1000 replicates and units of branch length are represented by number of nucleotide substitutions per site. As shown in Fig. 1, four clades were formed; clade-1 belongs to *R. toruloides* strains. Clade-2 includes *Yarrowia lipolytica.* Our lab isolate was grouped with *R. pacifica* strains in clade-3. Indicating evolutionary closeness to *R. pacifica*. Clade-4 comprised strains belonging to *R. mucilaginosa*. The ITS sequence was submitted in NCBI as *R. pacifica* INDKK with GenBank Accession No. MN560184. The evolutionary hierarchy of *Rhodotorula pacifica* INDKK was Eukaryota > Fungi > Dikarya > Basidiomycota > Pucciniomycotina > Microbotryomycetes > Sporidiobolales > Sporidiobolaceae > *Rhodotorula pacifica* INDKK.

### Selection of high triacylglycerol (TAG)-accumulating yeast isolates

**Fig. 2:**
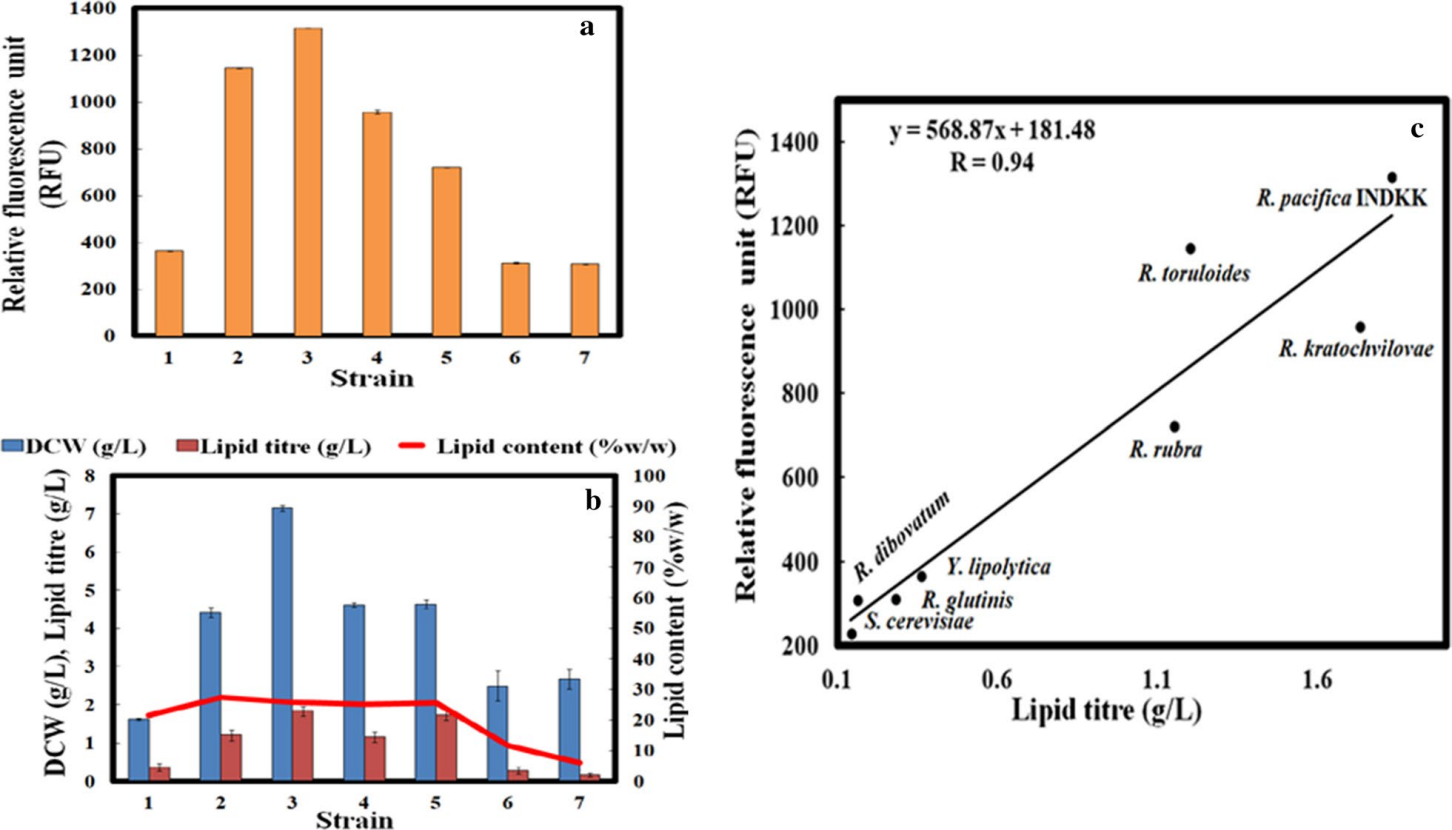
**a** RFU values of (1) *Y. lipolytica as control,* (2) *R. toruloides* (NCIM-3641), (3) *R. pacifica* INDKK, (4) *R. kratochvilovae* (MTCC-248), (5) *R. rubra* (NCIM-3260), (6) *R. glutinis* (NCIM-3168), (7)* R. dibovatum* (NCIM-3658) from Nile red spectrofluorimetry. **b** Comparison of DCW (g/L), lipid titre (g/L) and lipid content (% w/w) of six selected strains with (1) *Y. lipolytica as control,* (2) *R. toruloides* (NCIM-3641), (3)* R. pacifica* INDKK, (4) *R. kratochvilovae* (MTCC-248), (5)* R. rubra* (NCIM-3260), (6)* R. glutinis* (NCIM-3168), (7)* R. dibovatum* (NCIM-3658) after 72 h (one-way ANOVA test, *P* < 0.05). **c** Correlation plot showing liner correlation between lipid titres (g/L) from gravimetric analysis and RFU values of *S. cerevisiae*, *Y. lipolytica* (NCIM-3590), *R. toruloides* (NCIM-3641)*, R. pacifica* INDKK, *R. kratochvilovae *(MTCC-248), *R. rubra* (NCIM-3260), *R. glutinis* (NCIM-3168) and *R. dibovatum* (NCIM-3658)

Yeast isolates accumulating more than 20% TAG are considered as good candidates for microbial lipid-based fuel production [29]. For screening high lipid-accumulating strain, cells were grown in nitrogen (N)-limiting yeast nitrogen base (YNB) medium [30]. Initially, conventional Nile red staining-based spectrofluorimetry method [31] was tested with known high and low TAG accumulating yeast strains, *R. toruloides* (NCIM-3641) and *Saccharomyces cerevisiae*, respectively. Surprisingly, no emission was found at ~ 580 nm (corresponding to neutral lipids) and the emission at ~ 620 nm, (corresponding to polar lipids) was not able to distinguish between oleaginous and non-oleaginous yeast [32]. Hence, we optimized microwave-aided Nile red staining for yeast generating emission peak at ~ 580 nm and clearly differentiating the relative fluorescence unit (RFU) values of *R. toruloides* (NCIM-3641) and *S. cerevisiae* (Additional file 2: Figure S1). The 6 strains, *Rhodotorula pacifica* INDKK (lab isolate), *Rhodosporidium toruloides* (NCIM-3641)*, Rhodosporidium kratochvilovae* (MTCC-248)*, Rhodotorula rubra* (NCIM-3260)*, Rhodotorula glutinis* (NCIM-3168)*, Rhodosporidium dibovatum* (NCIM-3658) showed higher or comparable RFU value (Fig. 2a) to *Yarrowia lipolytica* (NCIM-3590), a previously reported high lipid-accumulating yeast isolate [33]. The high lipid accumulation in these isolates was further confirmed by lipid droplet (LD) size measurement using confocal microscopy (Additional file 3: Figure S2). DCW (g/L), lipid titre (g/L) and lipid content (%) of these isolates were also determined gravimetrically and *R. pacifica* INDKK showed maximum lipid titre (1.8 g/L) followed by *R. kratochvilovae* (1.6 g/L), *R. toruloides* (1.4 g/L) and *R. rubra* (1.2 g/L) at 72 h as shown in Fig. 2b. Gravimetric lipid analysis of the selected strains showed good correlation with microwave-aided Nile red staining (correlation coefficient *R* = 0.94), suggesting the reliability of microwave-aided Nile red spectrofluorimetry for different yeast species and genera (Fig. 2c). Moreover, quantitative analysis of lipid was performed by TLC. The chromatogram showed highest lipid accumulation by *R. pacifica* INDKK with TAG (67.92%), MAG (12.11%), DAG (10.29%) and FFA (9.67%). Notably, TAG content in *R. pacifica* INDKK was 1.46-fold higher than *Yarrowia lipolytica* (NCIM-3590) (Additional file 4: Figure S3)*.*

**Fig. 3:**
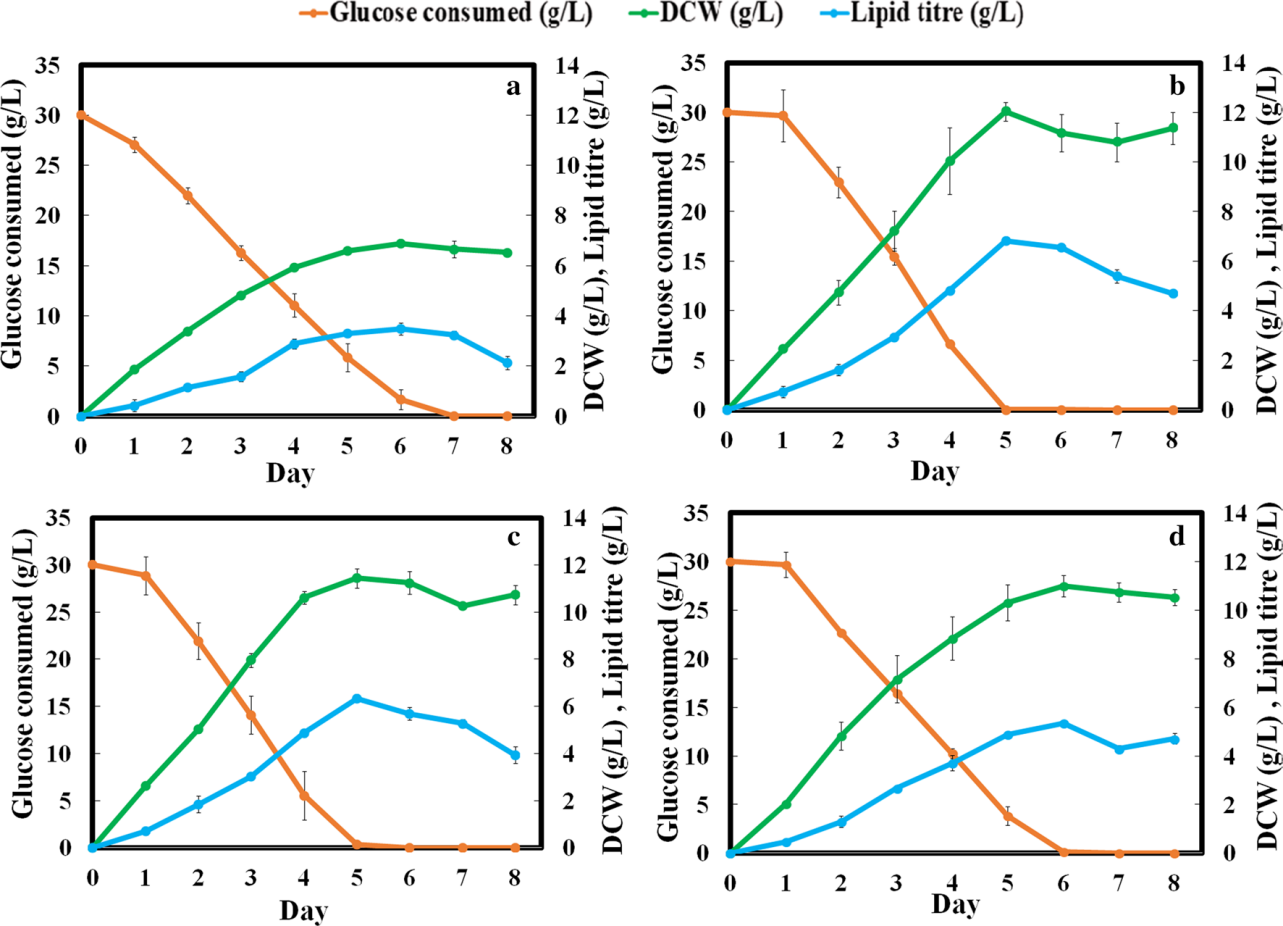
Time-course study of DCW, lipid titre and glucose consumption by selected oleaginous yeasts in YNB medium. **a** *R. toruloides* (NCIM-3641); **b** lab isolate *R. pacifica* INDKK; **c** *R. kratochvilovae* (MTCC-247); **d** *R. rubra* (NCIM-3560) (one-way ANOVA test, *P* < 0.05)

We further performed kinetic studies with four isolates showing > 20% w/w of lipid content in gravimetric analysis (*R. pacifica* INDKK*, R. toruloides, R. kratochvilovae, R. rubra)*. Kinetics of growth and lipid accumulation among these isolates revealed lowest DCW (6.88 ± 0.22 g/L), lipid titre (3.47 ± 0.05 g/L), lipid productivity (0.024 ± 0.05 g/L/h) and slow glucose utilization rate (0.17 g/L/h) by *R. toruloides* at 120 h (Fig. 3a). However, *R. pacifica* INDKK showed maximum DCW (12.8 ± 0.66 g/L), lipid titre (6.8 ± 0.4 g/L) and lipid productivity (0.056 ± 0.4 g/L/h) with glucose consumption rate of 0.25 g/L/h (Fig. 3b). Although glucose consumption rate (0.025 g/L/h) of *R. kratochvilovae* is similar to *R. pacifica* INDKK, it showed a lower lipid titre (6.32 g/L) **(**Fig. 3c). *R. rubra* also showed low lipid titre (5.35 g/L) and poor sugar consumption rate (0.02 g/L/h) as compared to *R. pacifica INDKK* (Fig. 3d). Together, among all the tested isolates *R. pacifica* INDKK showed maximum potential for lipid accumulation and was selected for further analysis in this study.

### *R. pacifica* INDKK assimilated wide range of sugars and displayed inhibitor-tolerant phenotypes

**Fig. 4:**
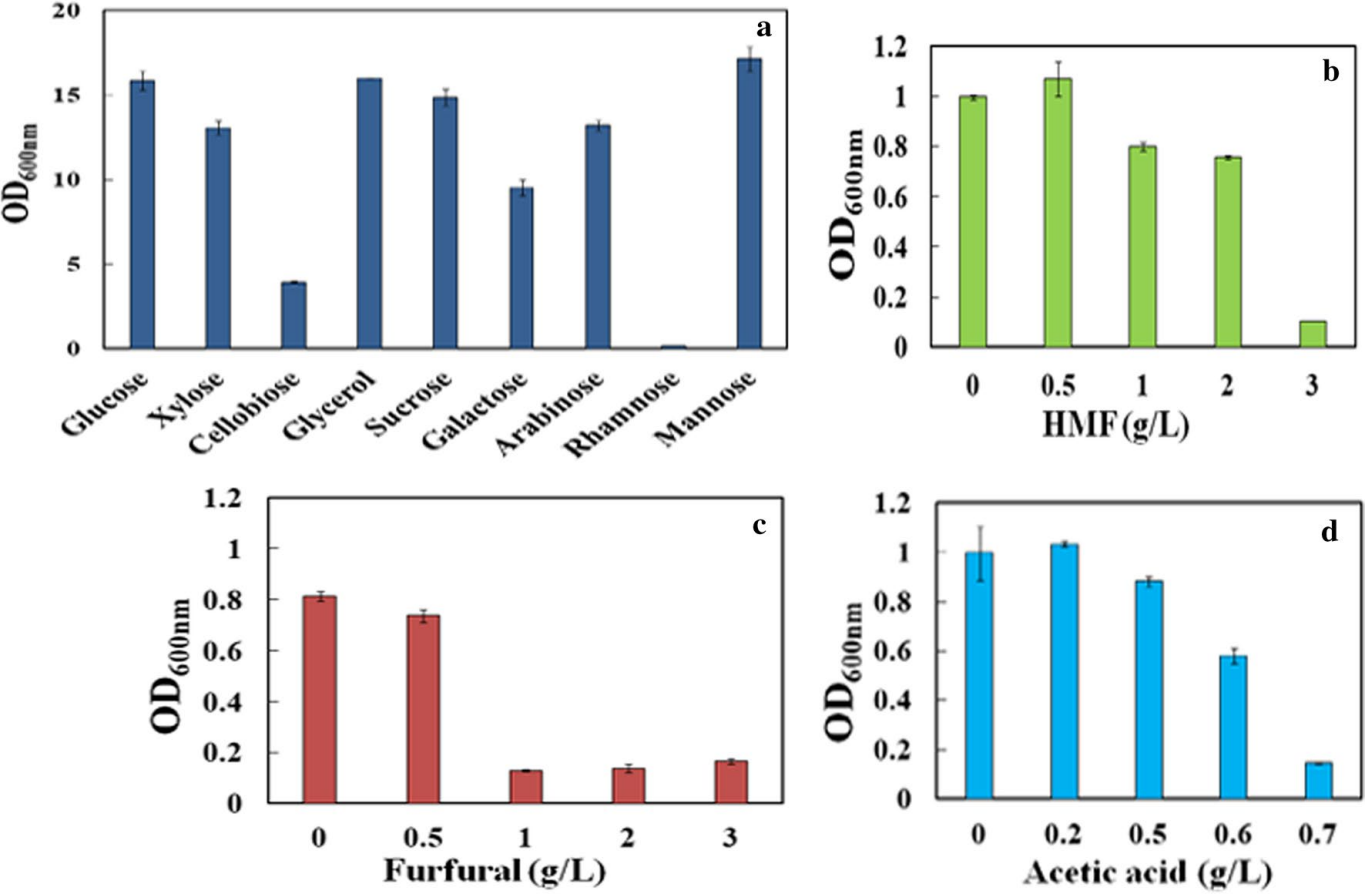
**a** Growth study of *R. pacifica* INDKK in different C6, C5 and C3 carbon sources and tolerance study in pre-treatment-generated inhibitors; **b** 5-HMF; **c** furfural and **d** acetic acid

Hydrolysates of lignocellulosic biomass contain mixture of C5 and C6 sugars and toxic inhibitors generated during pre-treatment such as furfural, acetic acid and 5-HMF [34]. These inhibitors reduce cell growth as well as lipid yield and productivity [35]. Therefore, yeast isolates capable of assimilating wide range of sugars (C5 and C6) along with enhanced tolerance to pre-treatment inhibitors are very important for economical microbial lipid production [36]. Interestingly, *R. pacifica INDKK* was able to grow on all the tested C6, C5 and C3 sugars except cellobiose and rhamnose (Fig. 4a). Moreover, the presence of lignocellulosic hydrolysate inhibitors such as 5-HMF (2 g/L), furfural (0.5 g/L) and acetic acid (0.5 g/L) in culture media did not show any significant effect on *R. pacifica INDKK* cell growth (Fig. 4b–d).

### Microbial lipid production using PS

Pongamia tree bears non-edible fruits whose shells after oil extraction from the seeds are generally discarded or burned [37]. Compositional analysis showed that PS contains 56.8% w/w holocellulose, 12% w/w cellulose and 8% w/w of xylan (Table 1). However, PS has not been considered as source of carbon and nitrogen for microbial cell growth thus far. In this regard, we explored hydrolysate of *Pongamia pinnata* shells for growth and lipid production by our newly isolated yeast isolate *R. pacifica INDKK*.

**Table 1:** The pongamia shell hydrolysate (PSH) nutrient composition before and after consumption by *Rhodotorula pacifica* INDKK

Component	PS (dry)	Before cultivation	After cultivation
Cellulose (%)	12	–	–
Holocellulose (%)	56.8	–	–
Xylan (%)	8	–	–
Glucose (g/L)	–	28.05 ± 0.01	–
Xylose (g/L)	–	18.13 ± 0.04	0.05 ± 0.08
Arabinose (g/L)	–	0.29 ± 0.01	–
Total proteins (g/L)	–	0.33 ± 0.02	0.23 ± 0.005
Total nitrogen (g/L)	1.90	0.053 ± 0.01	0.037 ± 0.02
Calcium (mg/L)	–	107 ± 0.87	6.3 ± 2.28
Sodium (g/L)	–	1.08 ± 0.15	0.84 ± 0.28
Magnesium (g/L)	–	1.53 ± 0.003	0.22 ± 0.06
Phosphorous (g/L)	2.05	1.51 ± 0.001	0.36 ± 0.12
Potassium (g/L)	6.32	1.53 ± 0.005	0.38 ± 0.12
Manganese (mg/L)	–	0.37 ± 0.05	0.24 ± 0.07
Iron (mg/L)	–	0.067 ± 0.009	0.036 ± 0.021
Sulphur (g/L)	0.276	–	–
5-HMF (g/L)	–	0.013 ± 0.005	0.01 ± 0.01
Acetic acid (g/L)	–	0.046 ± 0.01	–

– not detected.

**Fig. 5:**
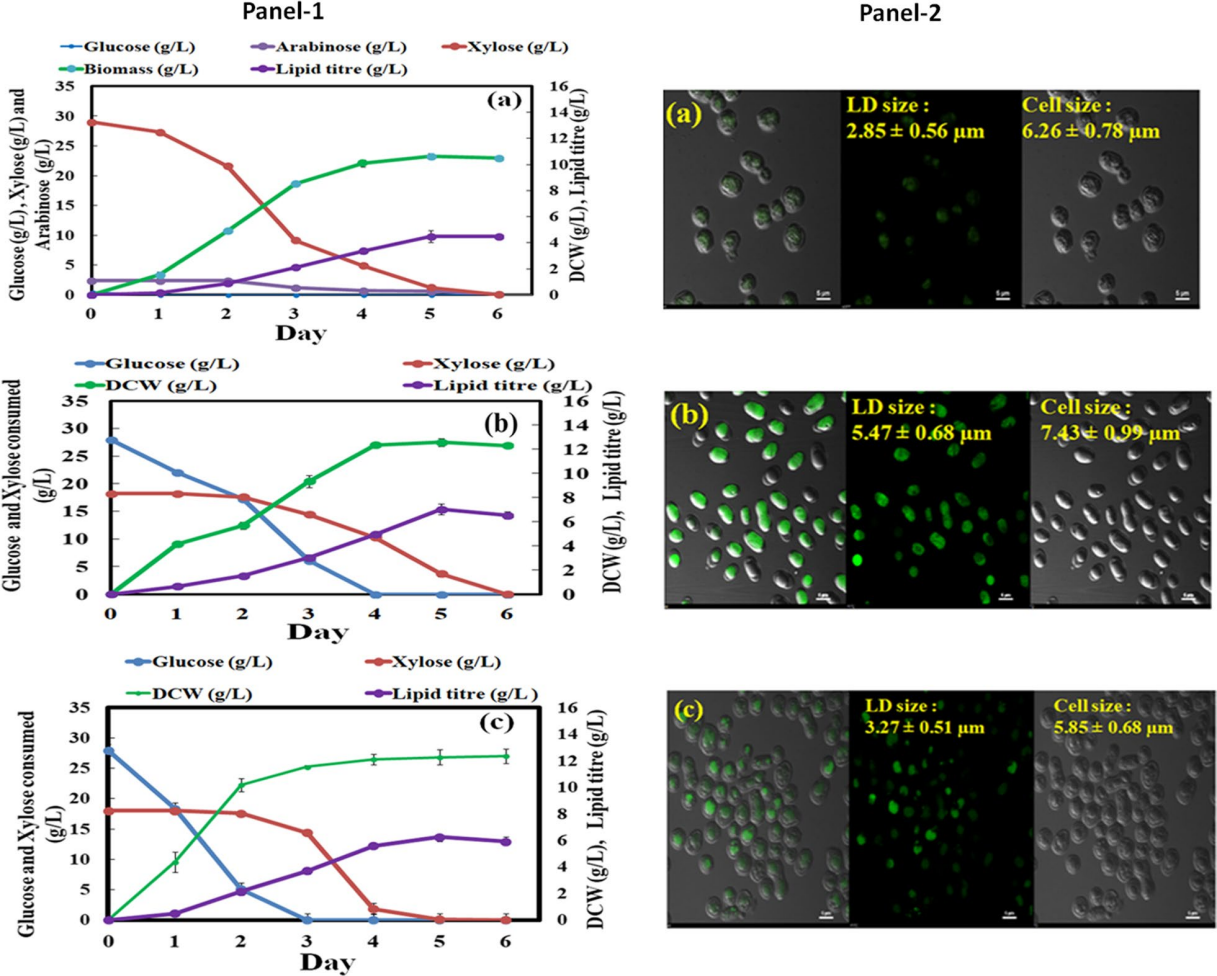
Panel: 1 Time-course study of DCW, lipid titre and sugar consumption by *R. pacifica* INDKK in **a** AD-PSH **b** AS-PSH and **c** control (YNB) (one-way ANOVA test, *P* < 0.05). Panel: 2 Confocal microscopy of *R. pacifica* INDKK cells cultivated in **a** AD-PSH, **b** AS-PSH, and **c** control (YNB) after 120 h showing average LD size and cell size at 100×

PS were subjected to acid as well as alkali pre-treatments (Additional file 5: Table S2). Liquid fraction of acid treatment showed higher sugar concentration (37.38 g/L) while alkali-treated liquid fraction obtained negligible amount of fermentable sugars (0.7 g/L total sugars). Therefore, liquid fraction of acid treatment was detoxified by activated charcoal, which reduced acetic acid concentration from 5.61 ± 0.035 g/L to 0.11 ± 0.005 g/L, completely removed furfural and 5-HMF with slight reduction in sugar concentration (14.63%). The acid-treated and detoxified liquid fraction of PSH (AD-PSH) contains 31.91 ± 0.042 g/L of total sugars (0.45 g/L glucose, 29.01 g/L xylose and 2.45 g/L arabinose). Time course study revealed that *R. pacifica* INDKK consumed all the sugars after 120 h of growth and produced 10.63 ± 0.004 g/L of DCW with 4.48 ± 0.02 g/L of lipid titre and 0.037 ± 0.001 g/L/h of lipid productivity (Fig. 5. Panel-1 5a).

The solid fraction of both acid-treated and alkali-treated PS were subjected to enzymatic hydrolysis (as described in “Methods”). The enzymatic saccharification of solid fraction from acid-treated PS yielded very less fermentable sugars (7.63 g/L) along with inhibitors such as acetic acid (0.3 g/L) and furfural (0.19 g/L) while enzymatic saccharification of solid fraction of alkali-treated PS resulted in high amount of total sugars (46.47 g/L), wherein glucose was the most abundant (28.05 ± 0.01 g/L) followed by xylose (18.13 ± 0.04 g/L) and arabinose (0.29 ± 0.01 g/L). As expected very low amount of inhibitors (5-HMF 0.013 ± 0.005 g/L, furfural 0 g/L and acetic acid 0.046 ± 0.01 g/L) was detected. Interestingly, trace elements such as Mn (0.37 ± 0.05 mg/L), K (1.53 ± 0.005 g/L), Ca (107 ± 0.87 mg/L), Fe (0.67 ± 0.009 mg/L), Na (1.08 ± 0.15 g/L) and Mg (1.53 ± 0.003 g/L) were also found in alkaline-treated saccharified PSH (AS-PSH), which could be helpful in cell growth and lipid accumulation. There was a drastic reduction (17-fold) in calcium ions which might be due to increase in calcium influx into the cell to cope up with the physiological stress conditions [38, 39]. Time course analysis of *R. pacifica* INDKK in AS-PSH showed that all the sugars (C6 and C5) were consumed after 120 h of growth and maximum 12.6 ± 0.5 g/L of DCW, 7.02 ± 0.7 g/L of lipid titre, 0.104 ± 0.004 g/L/h of biomass productivity and 0.058 ± 0.006 g/L/h of lipid productivity were achieved (Fig. 5, Panel-1 5b). Remarkably, the DCW and lipid productivities were 1.02 and 1.12-fold higher in AS-PSH as compared to YNB (0.101 ± 0.005 g/L/h of biomass productivity and 0.052 ± 0.003 g/L/h of lipid productivity) (Fig. 5, Panel-1 5c). Moreover, after 120 h of growth 30.0% reduction in protein content and 94.11, 22.22, 85.62, 73.71, 75.16 and 35.13% utilization of trace elements corresponding to Ca, Na, Mg, P, K and Mn were also observed. The lipid accumulation in PSH batch-cultivated cells was also confirmed by confocal microscopy. The average cell size (7.43 ± 0.99 µm) and average LD size (5.47 ± 0.68 µm) was 1.67 and 1.27-fold higher in AS-PSH cultivated cells as compared to YNB medium, respectively (Fig. 5, Panel-2)**.** However, cell size and LD size was 1.18 and 1.56-fold higher in AS-PSH grown cells as compared to AD-PSH (6.26 ± 0.78 and 3.49 ± 0.56 µm), respectively. Together, batch cultivation in AS-PSH significantly showed more biomass and lipid productivity as compared to AD-PSH (P value < 0.05).

### Fatty acid profile and analysis of biodiesel properties

The fatty acid profile of lipids extracted from *R. pacifica* INDKK cultivated in AS-PSH and YNB were analysed by gas chromatography–mass spectroscopy (GC–MS) after transesterification (as described in “Methods”). The 91.77% of FAME yield was achieved after transesterification with stearic (18:0), oleic (18:1), linoleic (18:2), linolenic (18:3) and palmitic (16:0) fatty acids, which is desirable for biodiesel production [40].

The lipids extracted from AS-PSH-grown *R. pacifica* INDKK showed higher amount of C18:1 (52.58%) followed by C16:0 (28.85%), C18:2 (12.45%), C18:3 (1.37%), C14:0 (0.89%), C22:0 (0.46%) and C15:0 (0.1%) fatty acids as depicted in Table 2. The fatty acid profile of *R. pacifica* INDKK grown in AD-PSH also showed higher C18:1 (61.98%) followed by C18:2 (16.86%), C16:0 (14.45%), C18:0 (6.13%) and C18:3 (1.37%). Interestingly, C18:2, C18:3 and C22:0 fatty acids were not detected in YNB-grown *R. pacifica* INDKK. Moreover, saturated fatty acid (SFA, 30.19%) and monounsaturated fatty acid (MUFA, 52.58%) were higher in AS-PSH-grown cells as compared to YNB (SFA 10.95% and MUFA 43.61%). The CN in AS-PSH and YNB were 57.29 and 86.86, respectively. The CN of *R. pacifica* INDKK grown in AS-PSH is higher as compared to AD-PSH (CN 53.92). The result showed that IV of AS-PSH (73.58 g I_2_/100 g) and YNB (39.02 g I_2_/100 g) grown cells were in accordance with the standards of EN 14214. Compared to rape seed oil and jatropha oil, *R. pacifica* INDKK oil in AS-PSH-grown cells contains more MUFA, SFA and less polyunsaturated fatty acid (PUFA), conferring greater CN. Other estimated biodiesel properties such as kinematic viscosity (KV, 3.7 mm^2^/s) and density (0.84 g/cm^3^) were comparable to rape seed oil, jatropha oil and standard values specified by EN 14214, ASTM D6751 and IS 15607 (Table 2). The above speculated biodiesel property values obtained from AS-PSH grown *R. pacifica* INDKK satisfies the specifications precisely by EN-14214, ASTM-D6751 and IS-15607 standards, suggesting it as an ideal biodiesel feedstock.

**Table 2:** Comparative FAME profile and biodiesel properties of *R. pacifica* INDKK cultivated in PSH and YNB

Fatty acid/biodiesel properties	Rape seed oil methyl ester [30]	Jatropha oil methyl ester [30]	AD-PSH	AS-PSH	Control (YNB)	EN 14214	ASTM D6751	IS 15607
C14:0 (%)	ND	ND	ND	0.89	0.55	–	–	–
C16:0 (%)	11.9	14.9	14.45	28.85	10.4	–	–	–
C16:1 (%)	ND	1	ND	ND	ND	–	–	–
C18:0 (%)	4.1	6.1	6.13	ND	ND	–	–	–
C18:1 (%)	20.8	40.4	61.98	52.58	43.61	–	–	–
C18:2 (%)	53.8	36.2	16.86	12.45	ND	–	–	–
C18:3 (%)	9.3	0.3	ND	1.37	ND	–	–	–
C20:0 (%)	ND	ND	ND	ND	ND	–	–	–
C22:0 (%)	ND	ND	ND	0.46	ND	–	–	–
C24:0 (%)	ND	ND	ND	ND	ND	–	–	–
Iodine value (IV) (g of I_2_/100 g)	107.76	98.02	86.29	73.58	39.02	120 (max.)	–	–
Cetane number (CN)	54.35	55.23	53.92	56.72	86.86	47	51	51
High heating value (HHV) (MJ/kg)	40.78	40.55	39.42	38.14	21.56	–	–	–
Density (g/cm^3^)	0.80	0.88	0.87	0.84	0.47	–	0.86–0.90	0.86–0.89
Kinematic viscosity (KV) (mm^2^/s)	4.4	4.48	3.90	3.7	2.13	1.9–6	3.5–5	2.5–6
Cold filter plugging point (CFPP)	ND	ND	−6.1	−5.97	−13.2	–	–	–

– No standard limit designated by biodiesel standards ASTM D6751, EN 14214 and IS 15607; *ND* not detected.

It was estimated that 6.8 g microbial lipid was obtained from 200 g of dry PS (20% w/v), from which 6.24 g of biodiesel was produced (Table 3) in this study.

**Table 3:** Biodiesel production from *R. pacifica* INDKK using PS as substrate

Pongamia shell powder used (%)	*R. pacifica* INDKK			
	DCW produced (g /L)	Lipids produced (g/L)	Biodiesel produced (g/L)	Transesterification efficiency (%)
20	12.8	6.8	6.24	91.77

## Discussion

Biodiesel-derived from lignocellulosic materials is often challenging and costly because of additional material processing steps such as biomass pre-treatment to release sugars and removal of pre-treatment-generated inhibitors in the hydrolysates that hinder fermentation. Therefore, use of potential oleaginous yeast that could simultaneously utilize mixed carbon sources and show tolerance to inhibitors will reduce the major obstacles of biodiesel production. Over the past few years many inhibitor-tolerant oleaginous yeast have been found, but their lipid production performances are still substandard. Hence, the quest for robust oleaginous yeast is still relevant. Yeast isolates isolated from sites of biomass degradation have shown great potential for TAG accumulation [41]. To untap the potential of oleaginous yeasts isolated from natural habitats related to lignocellulosic biodiesel production, 57 yeast isolates were screened.

High-throughput microwave-aided Nile red staining was found to be quick, effective and easy method for screening high TAG accumulating oleaginous yeasts as compared to other traditional methods for lipid estimation. This method clearly differentiates the RFU values between oleaginous and non-oleaginous yeast. Among 57 yeasts, 6 strains belonging to *Rhodotorula* and *Rhodosporidium* species showed higher RFU values. Further, gravimetric analysis data showed that four strains (*R. pacifica* INDKK*, R. toruloides, R. kratochvilovae, R. rubra*) displayed > 20% lipid content. Interestingly, when kinetic analysis was performed, our isolate *R. pacifica* INDKK displayed high lipid productivity with effective sugar utilization rate, which could be by stimulation of genes related to growth and lipid production [42]. Inhibitors present in lignocellulosic hydrolysates (acetic acid, 5-HMF, furfural, etc.) inhibit yeast growth. Acetic acid inhibits growth by repressing the expression of genes involved in nutrient transporters such as glucose transporters (HXT1 and HXT3) [43, 44]. 5-HMF inhibits dehydrogenases and glycolysis, whereas furfural reduces growth by inhibiting the key enzymes of carbon metabolism, increased production of radical oxygen species which damage DNA, protein and membranous structures [45].

Interestingly, increased growth was observed in 0.2 g/L concentration and displayed reduction of growth at 0.6 g/L of acetic acid. The increase in growth at 0.2 g/L could be due to the utilization of acetic acid as carbon source at this concentration. The yeast cells were also tolerant to 5-HMF (2 g/L) and furfural (0.5 g/L), beyond this concentration significant decrease in growth was observed. According to previously reported studies, most *Rhodosporidium* species could not tolerate furfural at 0.5 g/L concentration while *Rhodotorula glutinis* showed growth inhibition by 5-HMF > 0.5 g/L [36]. Therefore, in this study enhanced tolerance to inhibitors was observed by *R. pacifica INDKK.* It also showed ability to utilize all the tested C6, C5 and C3 sugars effectively. However, no significant growth was observed on rhamnose and cellobiose in comparison to glucose (*P* < 0.05). *R. pacifica INDKK* showed similar carbon source utilization profile as reported earlier in *Rhodosporidium mucilaginosa* [36]. The data elucidate that *R. pacifica INDKK* was tolerant to pre-treatment-generated inhibitors with potential to utilize different carbon sources present in lignocellulosic hydrolysates. The results were consistent with previously reported literature, wherein yeast such as *Trichosporon fermentans* and *Cryptococcus curvatus* were reported with similar potential but with low lipid productivity [6, 33].

Lignocellulosic lipid production is a multistep process where feedstock collection and valorization itself accounts for 70–80% biodiesel production cost. Therefore, we exploited abundantly available lignocellulosic waste PS as feedstock to reduce the major obstacle of biodiesel production using oleaginous yeast. Notably, when isolate *R. pacifica* INDKK was tested with AD-PSH and AS-PSH as carbon sources, we achieved lipid titre of 4.48 and 7.02 g/L at 30 °C in 120 h. The higher biomass and lipid was observed with AS-PSH, which could be due to preferable utilization of glucose as carbon source as compared to xylose. Also, AS-PSH contains more nutrients which supports the growth of *R. pacifica* INDKK. The gravimetric data were in co-relation to confocal microscopy study (*P* < 0.05). The lipid productivity of *R. pacifica* INDKK in AS-PSH (0.058 g/L/h) was higher than the previously reported oleaginous yeasts on different lignocellulosic hydrolysates such as 0.041 g/L/h on waste office paper enzymatic hydrolysate [18], 0.02 g/L/h on saccharified sweet sorghum juice [46, 47], and 0.029 g/L/h on corn stover enzymatic hydrolysate [48] as shown in Table 4. To the best of our knowledge, no yeast isolate reported has produced equivalent lipid titre to isolate *R. pacifica* INDKK on AS-PSH with effective glucose utilization rate.

**Table 4:** Comparative study of *R. pacifica* INDKK with different oleaginous yeasts grown in lignocellulosic biomass

Strain	Substrate	Biomass (g/L)	Lipid titre (g/L)	Lipid content (%)	Lipid productivity (g/L/h)	References
*Trichosporon fermentans*	Saccharified sweet sorghum	15.51	1.8	11.6	0.020	[46]
*Cryptococcus curvatus*	Acid treated and saccharified waste office paper hydrolysate	11.48	4.95	43.11	0.041	[18]
*Cryptococcus podzolicus* SCTCC30292	Ammonium carbonate-steam explosion pre-treated and saccharified corn stover	10.56	5.03	47.6	0.029	[48]
*Cryptococcus curvatus*	Lime treated and saccharified sorghum bagasse hydrolysate	6	2.6	43.3	0.021	[53]
*Rhodotorula pacifica* INDKK	YNB	12.26	6.61	54.24	0.055	This study
*Rhodotorula pacifica* INDKK	Acid treated and detoxified PSH	10.63	4.48	42.14	0.037	This study
*Rhodotorula pacifica* INDKK	Alkali treated and saccharified PSH	12.56	7.02	55.89	0.058	This study

Hoekman et al. [49] reported that differences in degree of unsaturation and carbon chain length influence the properties and performance of biodiesel. For better OS, biodiesel should have high amount of SFA and MUFA, but low amount of multi-unsaturated FAME. While biodiesel should have low amount of long-chain SFA for good low-temperature performance [49], the fatty acid composition of *R. pacifica* INDKK was rich in C16:0 and C18:1 depicting improved biodiesel properties such as CN and OS [50, 51]. The biodiesel obtained must meet the fuel standards (EN 14214, ASTM D6751, and IS) specifications before using it as a pure fuel. The CN parameter of diesel engine determines the auto-ignition quality of the fuel [49]. The high MUFA content elucidates balance between CFPP and OS for better quality of biodiesel [52]. The biodiesel properties obtained were in range of the biodiesel standard specifications illustrating the vehicular quality. The KV and density were similar to rape seed oil and jatropha oil. Results indicated that high CN of biodiesel from *R. pacifica* INDKK cultivated on AS-PSH significantly affects engine performance and start of injection with improved ignition characteristic. Longer OS and low CFPP properties of biodiesel obtained lead to longer shelf life and good engine performance, respectively. Hence, biodiesel production from *R. pacifica* INDKK cultivated on PSH was environmentally friendly and cost-efficient. Therefore, this process could be considered as an important step for the development of a cost-effective biodiesel production process.

## Conclusions

In this study, 57 yeast isolates were screened for lipid accumulation by microwave-aided Nile red spectrofluorimetry. Among them, novel oleaginous yeast isolate *R. pacifica* INDKK (Gen Bank accession No: MN560184) showed highest lipid accumulation, ability to grow on wide range of carbon sources and also displayed pre-treatment inhibitor-tolerant growth phenotypes. *R. pacifica* INDKK showed maximum DCW (12.8 ± 0.66 g/L), lipid titre (6.8 ± 0.4 g/L) and lipid productivity (0.056 ± 0.4 g/L/h) with glucose consumption rate of 0.25 g/L/h in YNB, among all the tested strains. *R. pacifica* INDKK can utilize wide range of C5 and C6 sugars and showed pre-treatment inhibitor-tolerant phenotypes. *R. pacifica* INDKK produced more DCW (12.56 ± 0.5 g/L), lipid titre (7.02 ± 0.4 g/L) and lipid productivity (0.058 ± 0.006 g/L) in AS-PSH as compared to AD-PSH (DCW, 10.63 ± 0.37 g/L; lipid titre, 4.48 ± 0.78 g/L; lipid productivity, 0.037 ± 0.006 g/L/h) and YNB (DCW, 12.26 ± 0.68 g/L; lipid titre, 6.65 ± 0.35 g/L; lipid productivity, 0.055 ± 0.003 g/L/h) grown cells. C18:1 (52.58%), C16:0 (28.85%) and C18:2 (12.45%) were major fatty acids detected in AS-PSH-grown cells, attributing better quality of the fuel. The present study demonstrates the lipogenic potential of *R. pacifica* INDKK for the production of biodiesel using PS as feasible feedstock.

## Methods and materials

### Media and other chemicals

PS were collected from International Centre for Genetic Engineering and Biotechnology (ICGEB), New Delhi campus. All analytical reagents and solvents (chloroform, methanol, n-hexane, diethyl ether, glacial acetic acid, H_2_SO_4_) were of high-performance liquid chromatography (HPLC) grade. Nile red (9-diethylamino-5-benzo[α] phenoxazinone), Bodipy 493–503 nm, heptadecanoic acid (internal standard) and FAME external standard (Supelco 37 component FAME mix) for GC–MS analysis were procured from Sigma (USA). Standard for thin-layer chromatography (TLC) (Triolein), sugars (glucose, xylose, arabinose) were procured from Hi-Media Laboratories (Mumbai, India). YNB, yeast extract and peptone were purchased from Difco (USA).

### Screening and identification of oleaginous yeast

#### Yeast strains

Soil sample was collected from the Coringa mangrove forest, Kakinada, Andhra Pradesh (16.83139°N–82.33667°E), India. 10^–1^ to 10^–6^ serial dilutions were made in sterilized (0.9%) saline and seeded onto YPD agar plates (peptone, 2%; glucose, 2%; yeast extract, 1%; agar, 2%) supplemented with chloramphenicol (35 µg/mL) and neomycin (50 µg/mL). Plates were incubated at 30 °C for 72 h and colonies with yeast-like morphology were further streaked on YPD agar plates for pure single cell culture. Yeast strains were also procured from National Collection of Industrial Microorganisms (NCIM), Pune, and Microbial Type Culture Collection and Gene Bank (MTCC), Chandigarh.

### Microwave-aided Nile red staining and screening of oleaginous yeast

Yeast isolates were screened for lipid accumulation by using microwave-assisted Nile red staining protocol [54] with modifications. Briefly, single colony culture of each strain was grown overnight in YPD medium at 30 °C and 200 rpm (pre-culture). The pre-culture was centrifuged and washed twice with Milli-Q (MQ) water, re-suspended in 100 mL YNB medium with glucose (3%) and (NH_4_)_2_SO_4_ (0.5%) to optical density (OD) of 0.2 at 600 nm and incubated at 30 °C for 3 days at 200 rpm. Cells corresponding to OD 1 of the above grown cultures were centrifuged (5000 × *g*, 4 min) and re-suspended in 50 µL of dimethyl sulphoxide (DMSO) followed by microwave treatment (1250 watts power for 60 s). Cells were mixed with Nile red solution (10 µg/mL) and again subjected to microwave treatment (1250 watts power, 60 s). Four replicates of each treatment were prepared and relative fluorescence intensity (RFU) was measured at exciting and emission wavelengths of 475 and ~ 580 nm, respectively. Relative neutral lipid content was represented as RFU of LD [55].

### ITS sequencing and phylogenetic analysis

The 18S rDNA sequence was PCR amplified from the genomic DNA by using ITS1-F (TCCGTAGGTGAACCTGCG) and ITS4-R (TCCTCCGCTTATTGATATGC) universal primers in a thermocycler (Eppendorf, Nexus GSX1, Germany) [56]. The reaction was carried out in 50 μL containing 1.0 μL of genomic DNA, 2.5 μL of forward primer, 2.5 μL of reverse primer, 1.0 μL of deoxyribonucleotide triphosphate (dNTP) mix, 5.0 μL of PCR Taq buffer, 0.5 μL of Taq DNA polymerase (G-Biosciences, USA) and 37.5 μL of sterile water. The PCR conditions: initial denaturation (95 °C for 5 min), 30 cycles of denaturation (95 °C for 30 s), annealing (52 °C for 30 s) and extension (72 °C for 1.0 min), and a final extension (72 °C for 5.0 min). The PCR products were cleaned up using Gene JET PCR Purification Kit (Thermo scientific, Lithuania) and sequenced (Invitrogen BioServices, India). The PCR-amplified DNA fragments were sequenced and used for BLAST (Basic Local Alignment Search Tool) analysis in NCBI (National Centre for Biotechnology Information) database and were aligned by ClustalW (a multiple sequence alignment tool). Phylogenetic tree was constructed by MEGA (molecular evolutionary genetics analysis) X software using sequences with shared similarity.

### Cell growth in presence of different carbon sources and pre-treatment inhibitors

To know the carbon source utilization efficiency by *R. pacifica* INDKS, the experiment was conducted on individual carbon sources. For this, cells were grown in 100 mL YNB medium supplemented with 2% carbon source individually (sucrose, cellobiose, glucose, mannose, galactose, rhamnose, arabinose, xylose and glycerol) and incubated at 30 °C, 200 rpm for 3 days. The inhibitor tolerance profile was tested by growing cells in YNB with 2% glucose supplemented with varying concentrations of 5-HMF (0.5 to 3 g/L), furfural (0.5 to 3 g/L) and acetic acid (0.2 to 0.7 g/L). YNB without inhibitors was used as control medium for cell growth.

### Biomass and lipid production on PSH

PS were washed thoroughly with water, dried in oven (60 °C for 48 h) and crushed in grinder. ~ 20 g of dried powder was subjected to dilute acid (2% v/v) as well as alkaline (2% v/v) treatments in autoclave at 121 °C for 90 min. The liquid fraction of acid treatment was detoxified by activated charcoal (15% w/v) at 30 °C for 3 h. Solid fraction of both acid and alkali treatments were neutralized and subjected to enzymatic saccharification using 20 FPU of cellulases/g (Sigma, USA) of biomass at 50 °C, 150 rpm for 72 h [57]. The hydrolysate was filtered and used as cultivation medium after supplementation of micro-nutrients such as NH_4_SO_4_ (0.5 g/L), MgSO_4_ (1.5 g/L), KH_2_PO_4_ (1.5 g/L). In parallel, YNB supplemented with 46.5 g/L of sugars (glucose 28 g/L, xylose 18.18 g/L, and arabinose 0.3 g/L) was used as control for cell growth and lipid accumulation. Lipid production was carried out in Erlenmeyer flasks (250 mL) containing 100 mL medium by adding overnight grown cells of OD 0.2 and incubating at 30 °C, 200 rpm for 196 h with initial pH of 6.8. Cells were harvested by centrifugation, washed with MQ water, lyophilized (Labconco, USA) and DCW (g/L) was determined. Lipids were extracted from lyophilized cells by using modified Bligh and Dyer method [58], lipid titre (g/L) was measured gravimetrically. Biomass productivity (g/L/h), lipid productivity (g/L/h) and lipid contents (%) were calculated as described previously [52].

### Lipid analysis

Lipid analysis was performed by confocal microscopy (Nikon, India) after staining the cells with Bodipy dye (0.5 µg/mL DMSO) [21]. Cell sizes and LD sizes were measured by using Nikon software. TAG analysis was performed on TLC plates (Merck, India) with triolein as standard in hexane: diethyl ether: acetic acid (85:15:1, v/v/v) solvent system. TLC plates were immersed in methanolic-MnCl_2_ solution, dried and heated at 120 °C (20 min) [59]. The TAG estimations were performed by using Image-J software.

### Analytical methods

Sugar (glucose, xylose, arabinose) and inhibitor (5-HMF, furfural and acetic acid) concentrations in PSH were determined by using HPLC (Agilent 1260 Series) equipped with Aminex HPX-87H column (Bio-Rad, USA) and refractive index (RI) detector. The mobile phase H_2_SO_4_ (4 mM) at a flow rate of 0.3 mL/min at column temperature of 40 °C and the sugar as well as inhibitor were quantified by dividing the peak area of the sample with the peak area of standard (1.0 g/L) at specific retention time [28]. Trace elements were determined by inductively coupled plasma-induced ion chromatography–mass spectroscopy (ICP-MS) analysis (Agilent 7900) using argon as carrier gas and sample flow rate was 2.0 mL/min with approximately 2.5 min total analysis time per sample. The samples were acidified with nitric acid to pH below 2.0 and filtered through a 0.45-μm pore diameter membrane filter. The calibration curves were prepared by diluting ICP multi-element standard solution, including the blank [52]. The protein concentration was estimated by Bradford method [60]. Holocellulose, cellulose and xylan content were determined by using standard Association of Official Analytical Chemists (AOAC) methods of analysis [61]. The dried PS powder was also subjected to energy dispersive X-ray (EDX) elemental analysis.

### Transesterification and GC–MS analysis

Transesterification was performed by previously described method [62] with some modifications. Briefly, lyophilized yeast cells and 6% methanolic-H_2_SO_4_ in 1:20 ratio were taken in teflon-sealed tube and heated at 80 °C for 1 h. FAMEs were extracted into hexane phase and analysed by GC–MS (7890A series) equipped with Omegawax (30 m × 0.25 mm ID, 0.25 µm thickness) and Agilent 7000 QQQ MS [63]. Identification and quantification of FAMEs were performed by NIST (National Institute of Standards and Technology) mass spectral database, AMDIS (Automated Mass Spectral Deconvolution and Identification System) and mass hunter software. Physical properties of biodiesel were computed by using previously reported experimental equations [49] and collated with rape seed oil methyl ester, jatropha oil methyl ester and to EN 14214, ASTM D6751 and Indian standards IS156907 [64].

### Statistical analysis

All experiments were performed in minimum three replicates. Average values with standard deviation were mentioned. One-way ANOVA test with post hoc analysis by Tukey’s test was performed using Microsoft Office Excel 2013 (Microsoft, USA) to analyse statistical significance of the results. Statistical differences at *P* ≤ 0.05 were considered as significant.

## Ethics approval and consent to participate

Not applicable.

## Consent for publication

Not applicable.

## Competing interests

The authors declare that they have no competing interests.

## Supplementary information

**Additional file 1: Table S1.** List of yeast strains screened in this study by microwave aided Nile red spectrofluorimetry and their corresponding Relative fluorescence intensity (RFU) values.

**Additional file 2: Figure S1.** Comparison between conventional and Microwave aided Nile red spectrofluorimetry.

**Additional file 3: Figure S2.** Confocal microscopy of selected 6 strains and *Y. lipolytica* (control) showing cell size and lipid droplet size stained with Bodipy.

**Additional file 4: Figure S3.** TLC And densitometric analysis of lipid extracts cultivated in YNB medium from selected 6 strains.

**Additional file 5: Table S2.** Optimisation of Pre-treatment and saccharification process for pongamia shell hydrolysate (PSH) preparation.

## Data Availability

All data generated or analysed during this study are included in this manuscript and its additional files.

## Abbreviations

PS: Pongamia shells
PSH: Pongamia shell hydrolysate
AS-PSH: Alkali-treated saccharified pongamia shell hydrolysate
AD-PSH: Acid-treated and detoxified pongamia shell hydrolysate
TAG: Triacylglycerol
LD: Lipid droplet
NCBI: National Centre for Biotechnology Information
BLAST: Basic Local Alignment Search Tool
FAME: Fatty acid methyl esters
5-HMF: 5-Hydroxyl methyl furfural
TLC: Thin-layer chromatography
NIST: National Institute of Standards and Technology
ASTM D6751: American Society for Testing and Materials
IS156907: Indian standards
EN 14214: European
AMDIS: Automated Mass Spectral Deconvolution and Identification System
YNB: Yeast nitrogen base
YPD: Yeast extract peptone dextrose
RFU: Relative fluorescence unit
HPLC: High-performance liquid chromatography
ICP-MS: Inductively coupled plasma-induced ion chromatography–mass spectroscopy
GC–MS: Gas chromatography–mass spectroscopy
MUFA: Monounsaturated fatty acid
PUFA: Polyunsaturated fatty acid
SFA: Saturated fatty acid
OS: Oxidative stability
CFPP: Cold filter plugging point
CN: Cetane number
HHV: High heating value
KV: Kinematic viscosity
IV: Iodine value

## References

[1] United Nations. World Population Prospects 2019. Dep. Econ. Soc. Aff. World Popul. Prospect. 2019. 2019.

[2] Jian XY, An XP, Li YD, Chen JH, Wang M, Zeng JB (2017). All plant oil derived epoxy thermosets with excellent comprehensive properties. Macromol Am Chem Soc.

[3] Goon DE, Abdul Kadir SHS, Latip NA, Rahim SA, Mazlan M (2019). Palm oil in lipid-based formulations and drug delivery systems. Biomolecules.

[4] Panchal TM, Patel A, Chauhan DD, Thomas M, Patel JV (2017). A methodological review on bio-lubricants from vegetable oil based resources. Renew Sustain Energy Rev.

[5] Veljković VB, Biberdžić MO, Banković-Ilić IB, Djalović IG, Tasić MB, Nježić ZB (2018). Biodiesel production from corn oil: a review. Renew Sustain Energy Rev.

[6] Anuar MR, Abdullah AZ (2016). Challenges in biodiesel industry with regards to feedstock, environmental, social and sustainability issues: a critical review. Renew Sustain Energy Rev.

[7] Cho HU, Park JM (2018). Biodiesel production by various oleaginous microorganisms from organic wastes. Bioresour Technol.

[8] Beopoulus A, Nicaud J-M (2012). Yeast: a new oil producer?. OCL.

[9] Prabhu AA, Gadela R, Bharali B, Deshavath NN, Dasu VV (2019). Development of high biomass and lipid yielding medium for newly isolated *Rhodotorula mucilaginosa*. Fuel.

[10] Shi S, Zhao H (2017). Metabolic engineering of oleaginous yeasts for production of fuels and chemicals. Front Microbiol.

[11] Masri MA, Garbe D, Mehlmer N, Brück TB (2019). A sustainable, high-performance process for the economic production of waste-free microbial oils that can replace plant-based equivalents. Energy Environ Sci..

[12] Martínez EJ, Raghavan V, González-Andrés F, Gómez X (2015). New biofuel alternatives: Integrating waste management and single cell oil production. Int J Mol Sci.

[13] Park GW, Chang HN, Jung K, Seo C, Kim Y-C, Choi JH (2017). Production of microbial lipid by *Cryptococcus curvatus* on rice straw hydrolysates. Process Biochem Elsevier.

[14] Chen X-F, Huang C, Xiong L, Wang B, Qia G-X, Lin X-Q (2016). Use of elephant grass (*Pennisetum purpureum*) acid hydrolysate for microbial oil production by *Trichosporon cutaneum*. Prep Biochem Biotechnol.

[15] Brar KK, Sarma AK, Aslam M, Polikarpov I, Chadha BS (2017). Potential of oleaginous yeast *Trichosporon* sp., for conversion of sugarcane bagasse hydrolysate into biodiesel. Bioresour Technol..

[16] Deeba F, Pruthi V, Negi YS (2017). Fostering triacylglycerol accumulation in novel oleaginous yeast *Cryptococcus psychrotolerans* IITRFD utilizing groundnut shell for improved biodiesel production. Bioresour Technol.

[17] Liu Z, Feist AM, Dragone G, Mussatto SI (2020). Lipid and carotenoid production from wheat straw hydrolysates by different oleaginous yeasts. J Clean Prod..

[18] Nair AS, Al-Bahry S, Gathergood N, Tripathi BN, Sivakumar N (2020). Production of microbial lipids from optimized waste office paper hydrolysate, lipid profiling and prediction of biodiesel properties. Renew Energy.

[19] Ayadi I, Belghith H, Gargouri A, Guerfali M (2019). Utilization of wheat bran acid hydrolysate by *Rhodotorula mucilaginosa* Y-MG1 for microbial lipid production as feedstock for biodiesel synthesis. Biomed Res Int.

[20] Zhang Y, Xia C, Lu M, Tu M (2018). Effect of overliming and activated carbon detoxification on inhibitors removal and butanol fermentation of poplar prehydrolysates. Biotechnol Biofuels.

[21] Patel A, Sindhu DK, Arora N, Singh RP, Pruthi V, Pruthi PA (2015). Biodiesel production from non-edible lignocellulosic biomass of *Cassia fistula* L. fruit pulp using oleaginous yeast *Rhodosporidium kratochvilovae* HIMPA1. Bioresour Technol..

[22] Huang C, Cui XX, Wu H, Lou WY, Zong MH (2014). The effect of different factors on microbial oil production by *Trichosporon fermentans* on rice straw acid hydrolysate. Int J Green Energy.

[23] Pongamia Pinnata. https://www.jatrophaworld.org/pongamia_pinnata_84.html

[24] Ujjinappa S, Sreepathi LK (2018). Production and quality testing of fuel briquettes made from pongamia and tamarind shell. Sadhana.

[25] Amin A (2019). Review of diesel production from renewable resources: catalysis, process kinetics and technologies. Ain Shams Eng J.

[26] Patel A, Arora N, Mehtani J, Pruthi V, Pruthi PA (2017). Assessment of fuel properties on the basis of fatty acid pro fi les of oleaginous yeast for potential biodiesel production. Renew Sustain Energy Rev.

[27] Tamura K, Peterson D, Peterson N, Stecher G, Nei M, Kumar S (2011). MEGA5: molecular evolutionary genetics analysis using maximum likelihood, evolutionary distance, and maximum parsimony methods. Mol Biol Evol Narnia.

[28] Pandey AK, Kumar M, Kumari S, Kumari P, Yusuf F, Jakeer S (2019). Evaluation of divergent yeast genera for fermentation-associated stresses and identification of a robust sugarcane distillery waste isolate *Saccharomyces cerevisiae* NGY10 for lignocellulosic ethanol production in SHF and SSF. Biotechnol Biofuels..

[29] Jin M, Slininger PJ, Dien BS, Waghmode S, Moser BR, Orjuela A (2015). Microbial lipid-based lignocellulosic biorefinery: feasibility and challenges. Trends Biotechnol.

[30] Tanimura A, Takashima M, Sugita T, Endoh R, Kikukawa M, Yamaguchi S (2014). Selection of oleaginous yeasts with high lipid productivity for practical biodiesel production. Bioresour Technol.

[31] Sitepu IR, Ignatia L, Franz AK, Wong DM, Faulina SA, Tsui M (2012). An improved high-throughput Nile red fluorescence assay for estimating intracellular lipids in a variety of yeast species. J Microbiol Methods NIH Public Access.

[32] Alonzo F, Mayzaud P (1999). Spectrofluorometric quantification of neutral and polar lipids in zooplankton using Nile red. Mar Chem.

[33] Pawar PP, Odaneth AA, Vadgama RN, Lali AM (2019). Simultaneous lipid biosynthesis and recovery for oleaginous yeast *Yarrowia lipolytica*. Biotechnol Biofuels.

[34] Palmqvist E, Hahn-Hägerdal B (2000). Fermentation of lignocellulosic hydrolysates. II: inhibitors and mechanisms of inhibition. Bioresour Technol.

[35] Galafassi S, Cucchetti D, Pizza F, Franzosi G, Bianchi D, Compagno C (2012). Lipid production for second generation biodiesel by the oleaginous yeast *Rhodotorula graminis*. Bioresour Technol Elsevier.

[36] Sitepu I, Selby T, Lin T, Zhu S, Boundy-Mills K (2014). Carbon source utilization and inhibitor tolerance of 45 oleaginous yeast species. J Ind Microbiol Biotechnol..

[37] Halder PK, Paul N, Beg MRA (2014). Prospect of *Pongamia pinnata* (Karanja) in Bangladesh: a sustainable source of liquid fuel. J Renew Energy.

[38] Liu S, Hou Y, Liu W, Lu C, Wang W, Sun S (2015). Components of the calcium-calcineurin signaling pathway in fungal cells and their potential as antifungal targets. Eukaryot Cell Am Soc Microbiol.

[39] Popa CV, Dumitru I, Ruta LL, Danet AF, Farcasanu IC (2010). Exogenous oxidative stress induces Ca2+ release in the yeast *Saccharomyces cerevisiae*. FEBS J.

[40] Knothe G (2009). Improving biodiesel fuel properties by modifying fatty ester composition. Energy Environ Sci.

[41] Hoondee P, Wattanagonniyom T, Weeraphan T, Tanasupawat S, Savarajara A (2019). Occurrence of oleaginous yeast from mangrove forest in Thailand. World J Microbiol Biotechnol.

[42] Broach JR (2012). Nutritional control of growth and development in yeast. Genetics.

[43] Ding J, Bierma J, Smith MR, Poliner E, Wolfe C, Hadduck AN (2013). Acetic acid inhibits nutrient uptake in *Saccharomyces cerevisiae*: auxotrophy confounds the use of yeast deletion libraries for strain improvement. Appl Microbiol Biotechnol.

[44] Kitanovic A, Bonowski F, Heigwer F, Ruoff P, Kitanovic I, Ungewiss C (2012). Acetic acid treatment in *S. cerevisiae* creates significant energy deficiency and nutrient starvation that is dependent on the activity of the mitochondrial transcriptional complex Hap2-3-4-5. Front Oncol.

[45] Field SJ, Ryden P, Wilson D, James SA, Roberts IN, Richardson DJ (2015). Identification of furfural resistant strains of *Saccharomyces cerevisiae* and *Saccharomyces paradoxus* from a collection of environmental and industrial isolates. Biotechnol Biofuels..

[46] Antonopoulou I, Spanopoulos A, Matsakas L (2020). Single cell oil and ethanol production by the oleaginous yeast *Trichosporon fermentans* utilizing dried sweet sorghum stalks. Renew Energy.

[47] Liang Y, Tang T, Umagiliyage AL, Siddaramu T, McCarroll M, Choudhary R (2012). Utilization of sorghum bagasse hydrolysates for producing microbial lipids. Appl Energy.

[48] Xu H, Zhao N, Yao H, Qin H, Zeng J, Ran Y (2019). Lipid production from corn stover by a cost-efficient system featuring ammonium carbonate-steam explosion and recirculating enzymatic hydrolysis. Biomass Bioenergy Pergamon.

[49] Hoekman SK, Broch A, Robbins C, Ceniceros E, Natarajan M (2012). Review of biodiesel composition, properties, and specifications. Renew Sustain Energy Rev.

[50] Knothe G. Designer biodiesel: optimizing fatty ester composition to improve fuel properties. Energy and Fuels. 2008;22:1358–64. https://pubs.acs.org/sharingguidelines

[51] Knothe G (2011). The potential of biodiesel with improved properties to an alternative energy mix. Green Energy Technol.

[52] Deeba F, Patel A, Arora N, Pruthi V, Pruthi PA, Negi YS (2018). Amaranth seeds (*Amaranthus palmeri* L.) as novel feedstock for biodiesel production by oleaginous yeast. Environ Sci Pollut Res..

[53] Liang Y, Tang T, Siddaramu T, Choudhary R, Umagiliyage AL (2012). Lipid production from sweet sorghum bagasse through yeast fermentation. Renew Energy Pergamon.

[54] Chen W, Sommerfeld M, Hu Q (2011). Microwave-assisted Nile red method for in vivo quantification of neutral lipids in microalgae. Bioresour Technol Elsevier.

[55] Wang X, Balamurugan S, Liu S-F, Zhang M-M, Yang W-D, Liu J-S (2020). Enhanced polyunsaturated fatty acid production using food wastes and biofuels byproducts by an evolved strain of *Phaeodactylum tricornutum*. Bioresour Technol..

[56] Vasdinyei R, Deák T (2003). Characterization of yeast isolates originating from Hungarian dairy products using traditional and molecular identification techniques. Int J Food Microbiol.

[57] Sahoo D, Ummalyma SB, Okram AK, Pandey A, Sankar M, Sukumaran RK (2018). Effect of dilute acid pretreatment of wild rice grass (*Zizania latifolia*) from Loktak Lake for enzymatic hydrolysis. Bioresour Technol.

[58] Bligh EG, Dyer WJ (1959). A rapid method of total lipid extraction and purification. Can J Biochem Physiol.

[59] Deeba F, Pruthi V, Negi YS (2016). Converting paper mill sludge into neutral lipids by oleaginous yeast *Cryptococcus vishniaccii* for biodiesel production. Bioresour Technol.

[60] Bradford MM (1976). A rapid and sensitive method for the quantitation of microgram quantities of protein utilizing the principle of protein-dye binding. Anal Biochem.

[61] Sluiter JB, Ruiz RO, Scarlata CJ, Sluiter AD, Templeton DW (2010). Compositional analysis of lignocellulosic feedstocks. 1. Review and description of methods. J Agric Food Chem..

[62] Liu B, Zhao Z (2007). Biodiesel production by direct methanolysis of oleaginous microbial biomass. J Chem Technol Biotechnol..

[63] Liang Y, Cui Y, Trushenski J, Blackburn JW (2010). Converting crude glycerol derived from yellow grease to lipids through yeast fermentation. Bioresour Technol Elsevier.

[64] Deeba F, Pruthi V, Negi YS (2018). Aromatic hydrocarbon biodegradation activates neutral lipid biosynthesis in oleaginous yeast. Bioresour Technol.

