# Supplementary material for: Harnessing pongamia shell hydrolysate for triacylglycerol agglomeration by novel oleaginous yeast Rhodotorula pacifica INDKK
Source: Biotechnol Biofuels. 2020 Oct 19;13:175. doi: 10.1186/s13068-020-01814-9 (PMC7574204; doi:10.1186/s13068-020-01814-9)
Supplement: Supplementary file 1 — Additional file 1: Table S1. List of yeast strains screened in this study by microwave aided Nile red spectrofluorimetry and their corresponding Relative fluorescence intensity (RFU) values. [file 13068_2020_1814_MOESM1_ESM.doc]

**Additional file -1**

**Table S1. List of yeast strains screened in this study by microwave aided nile red spectrofluorimetry.**

| ***S.no*** | ***Strain*** | ***RFU**** | ***S.no*** | ***Strain*** | ***RFU**** |
| --- | --- | --- | --- | --- | --- |
| ***1*** | ***Saccharomyces cerevisiae-NG1, (Sugar distillery waste, Bijnor, Uttar pradesh,India) (Lab isolate)*** | ***284.12*** | ***30*** | ***Saccharomyces cerevisiae***  ***(NCIM-3219)*** | ***174.43*** |
| ***2*** | ***Kluyveromyces marxianus (Mother dairy, New delhi, India)(Lab isolate)*** | ***85.17*** | ***31*** | ***Yarrrowia lipolytica***  ***( NCIM- 3590)*** | ***365.23*** |
| ***3*** | ***Pichia kudriavzevii-NG19 (Brewery boiler, Bulandhshahar,Uttar Pradesh, India)***  ***(Lab isolate)***   \|  \| \| --- \| | ***32.8*** | ***32*** | ***Yarrrowia lipolytica***  ***( NCIM-3472)*** | ***121.54*** |
| ***4*** | ***Wikerhamomyces anomalus-NG25(Suagr distillery waste, Bijnor, Uttar Pradesh, India)***  ***(Lab isolate)*** | ***60.48*** | ***33*** | ***Torulospora delbrueckii***  ***(MTCC-2893)*** | ***202.7*** |
| ***5*** | ***Ogataea thermophile-NG10 (Sugarcane distillery waste, Bulandshahr, Uttar pradesh, India)***  ***(Lab isolate)*** | ***27.35*** | ***34*** | ***Torulospora delbruecki***  ***(MTCC-3415)*** | ***41.89*** |
| ***6*** | ***Candida tropicalis -NG 26,* (Sugarcane bagasse distillery waste, Bijnor, Uttar Pradesh, India) ( Lab isolate)**   \|  \| \| --- \| | ***68.69*** | ***35*** | ***Scheffersomyces spartinae***  ***(MTCC-9717)*** | ***31.23*** |
| ***7*** | ***NG-45* (Sugarcane bagasse distillery waste, Bijnor, Uttar Pradesh, India)( Lab isolate) *(Lab isolate)*** | ***64.23*** | ***36*** | ***Rhodosporidium paludigenum***  ***(MTCC-2889)*** | ***112.28*** |
| ***8*** | ***Candida lusitaniae (NCIM -3484)*** | ***31.63*** | ***37*** | ***Pichia Manshurica saito***  ***(MTCC-4799)*** | ***122.54*** |
| ***9*** | ***Kluyveromyces lactics (NCIM-3551)*** | ***83.19*** | ***38*** | ***Pichia Manshurica saito***  ***(MTCC-4051)*** | ***63.48*** |
| ***10*** | ***Candida shehatae (NCIM-3500)*** | ***103.29*** | ***39*** | ***Rhodosporidium paludigenum***  ***(MTCC-2890)*** | ***61.89*** |
| ***11*** | ***Pichia stipites (NCIM-3507)*** | ***53.21*** | ***40*** | ***Rhodosporidum kratochvilovae***  ***(MTCC-190)*** | ***248.16*** |
| ***12*** | ***Schwanniomyces occidentalis (NCIM-3424)*** | ***102.19*** | ***41*** | ***Rhodosporidum kratochvilovae***  ***(MTCC-247)*** | ***957.23*** |
| ***13*** | ***Trichosporan pullulans***  ***(NCIM-3151)*** | ***226.43*** | ***42*** | ***Starmerella bombicola***  ***(MTCC-1910)*** | ***31.76*** |
| ***14*** | ***Rhodosporidium toruloides***  ***(NCIM-3547)*** | ***136.76*** | ***43*** | ***Lipomyces starkeyi***  ***(MTCC-1400)*** | ***236.96*** |
| ***15*** | ***Rhodosporidium toruloides***  ***(NCIM-3641)*** | ***1144.3*** | ***44*** | ***Yarrowia lipolytica***  ***(MTCC-9517)*** | ***47.03*** |
| ***16*** | ***Rhodosporidium dibovatum***  ***(NCIM-3657)*** | ***276.01*** | ***45*** | ***Cryptococcus curvatus***  ***(MTCC-2698)*** | ***145.98*** |
| ***17*** | ***Rhodosporidium dibovatum***  ***(NCIM-3658)*** | ***307.22*** | ***46*** | ***Cryptococcus albidus***  ***(MTCC-2661)*** | ***208.81*** |
| ***18*** | ***Rhodotorula glutinis***  ***(NCIM-3168)*** | ***160.03*** | ***47*** | ***Blastobotrys adeninovorans***  ***(MTCC-2517)*** | ***263.12*** |
| ***19*** | ***Rhodotorula glutinis***  ***(NCIM-3169)*** | ***310.54*** | ***48*** | ***Coringa soil isolate (KS-1)*** | ***148.63*** |
| ***20*** | ***Rhodotorula minuta***  ***(NCIM-3359)*** | ***83.75*** | ***49*** | ***Coringa soil isolate (KS-2)***  ***(Lab isolate)*** | ***82.75*** |
| ***21*** | ***Rhodotorula minuta***  ***(NCIM-3427)*** | ***58.92*** | ***50*** | ***Coringa soil isolate (KS-3) (Lab isolate)*** | ***126.58*** |
| ***22*** | ***Pichia anomala***  ***(NCIM-3341)*** | ***205.65*** | ***51*** | ***Coringa soil isolate (KS-4) ( R. pacifica INDKK)*** | ***1316.1*** |
| ***23*** | ***Hansenula beijernikii***  ***(NCIM-3343)*** | ***76.8*** | ***52*** | ***Coringa soil isolate (KS-5)*** | ***40.90*** |
| ***24*** | ***Rhodotorula rubra***  ***(NCIM-3260)*** | ***721.02*** | ***53*** | ***Coringa soil isolate (KS-6)*** | ***45.37*** |
| ***25*** | ***Hansenula californica(NCIM-3438)*** | ***106.69*** | ***54*** | ***Coringa soil isolate (KS-7)*** | ***96.12*** |
| ***26*** | ***Cryptococcus laurentii(NCIM-3373)*** | ***38.69*** | ***55*** | ***Coringa soil isolate (KS-8)*** | ***24.06*** |
| ***27*** | ***Candida tropicali(NCIM-3121)*** | ***136.52*** | ***56*** | ***Coringa soil isolate (KS-9)*** | ***135.3*** |
| ***28*** | ***Candida magnolia(NCIM-3646)*** | ***216.6*** | ***57*** | ***Coringa soil isolate (KS-10)*** | ***115.41*** |
| ***29*** | ***Candida guilliermondii(NCIM-3124)*** | ***78.66*** |  |  |  |

**** RFU:* Relative fluorescence intensity (RFU) values estimated in this study by microwave aided nile red spectrofluorimetry.**
