# Supplementary material for: Harnessing pongamia shell hydrolysate for triacylglycerol agglomeration by novel oleaginous yeast Rhodotorula pacifica INDKK
Source: Biotechnol Biofuels. 2020 Oct 19;13:175. doi: 10.1186/s13068-020-01814-9 (PMC7574204; doi:10.1186/s13068-020-01814-9)

**Additional file - 2**

**Figure S1.**

1. **Conventional Nile red spectrofluorimetry showing emission peaks at ~ 620nm for both *R. toruloides* (NCIM -3641) (high lipid) strain and *Saccharomyces cerevisiae* (low lipid) strain.**
2. **Microwave aided Nile red spectrofluorimetry showing emission peaks at ~580 nm for both *R. toruloides* (NCIM -3641) (high lipid) strain and *Saccharomyces cerevisiae* (low lipid) strain.**


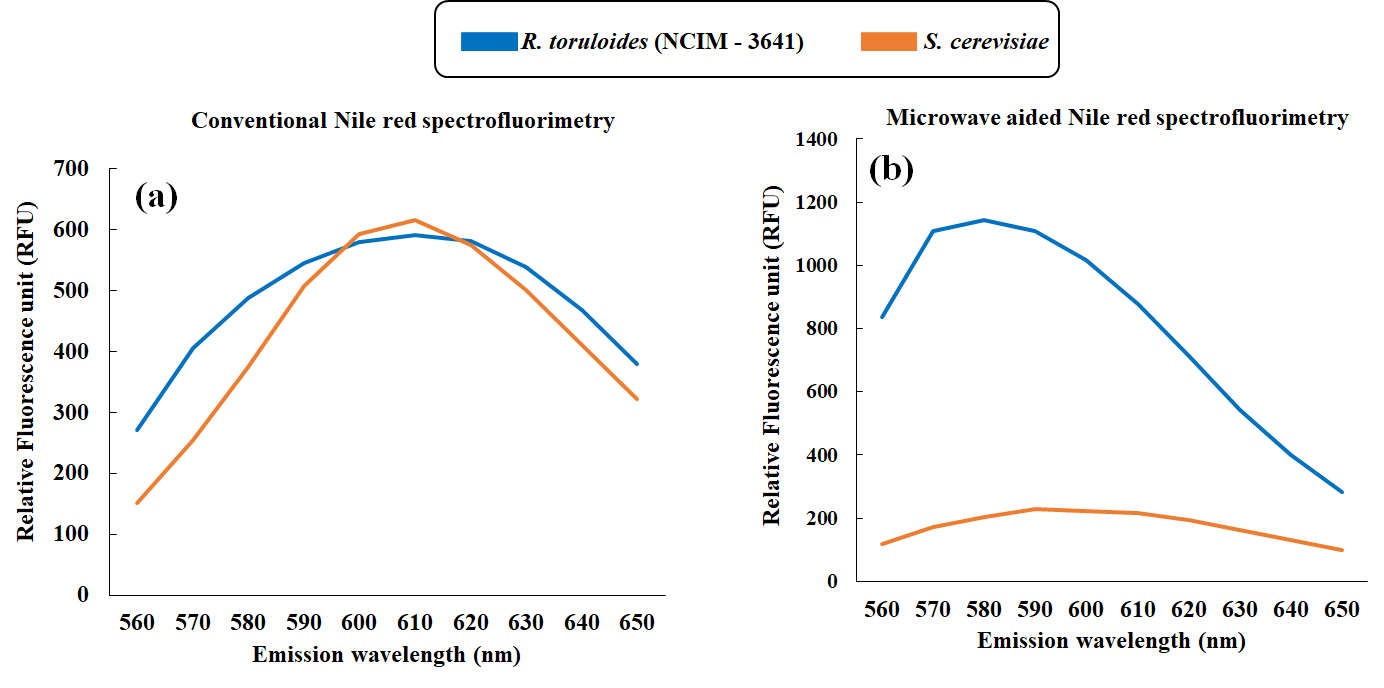

Supplement: Supplementary file 2 — Additional file 2: Figure S1. Comparison between conventional and Microwave aided Nile red spectrofluorimetry. [file 13068_2020_1814_MOESM2_ESM.doc]
