# Supplementary material for: Harnessing pongamia shell hydrolysate for triacylglycerol agglomeration by novel oleaginous yeast Rhodotorula pacifica INDKK
Source: Biotechnol Biofuels. 2020 Oct 19;13:175. doi: 10.1186/s13068-020-01814-9 (PMC7574204; doi:10.1186/s13068-020-01814-9)

**Additional file -3**

**Figure S2.**

**Confocal microscopy of (a) (Control)*Y. lipolytica* (NCIM-3590), (b) *R. toruloides* (NCIM-3641) *(c) R. pacifica* INDKK (d) *R. kratochvilovae* (MTCC-248) *(e) R. rubra* (NCIM-3260) *(f) R. glutinis* (NCIM-3168) *(g) R. dibovatum* (NCIM-3658) showing cell size and lipid droplet size.**


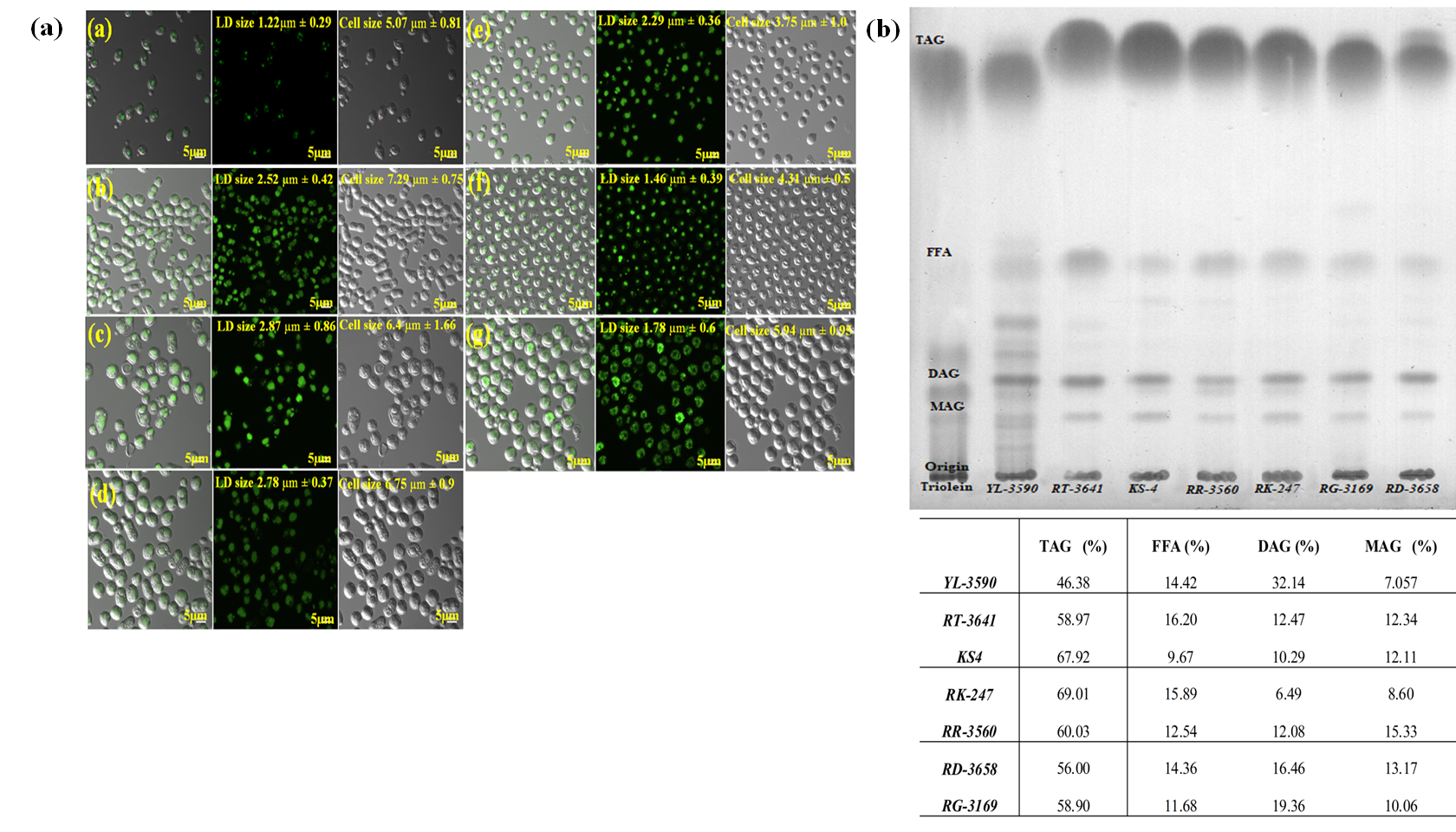

Supplement: Supplementary file 3 — Additional file 3: Figure S2. Confocal microscopy of selected 6 strains and Y. lipolytica (control) showing cell size and lipid droplet size stained with Bodipy. [file 13068_2020_1814_MOESM3_ESM.doc]
