# Supplementary material for: Harnessing pongamia shell hydrolysate for triacylglycerol agglomeration by novel oleaginous yeast Rhodotorula pacifica INDKK
Source: Biotechnol Biofuels. 2020 Oct 19;13:175. doi: 10.1186/s13068-020-01814-9 (PMC7574204; doi:10.1186/s13068-020-01814-9)
Supplement: Supplementary file 4 — Additional file 4: Figure S3. TLC And densitometric analysis of lipid extracts cultivated in YNB medium from selected 6 strains. [file 13068_2020_1814_MOESM4_ESM.doc]

**Additional file -4**

**Figure S3.**

**TLC analysis Control (Triloein), (a) *Y. lipolytica* (NCIM-3590), (b) *R. toruloides* (NCIM-3641) *(c) R. pacifica* INDKK (d) *R. kratochvilovae* (MTCC-248) *(e) R. rubra* (NCIM-3260) *(f) R. glutinis* (NCIM-3168) *(g) R. dibovatum* (NCIM-3658). Densitometric analysis of TLC revealed major % triacylglycerols (TAG) but low % of free fatty acids (FFA), diacylglycerols (DAG), monoacylglycerols (MAG) in all lipid extracts cultivated in YNB medium**

**
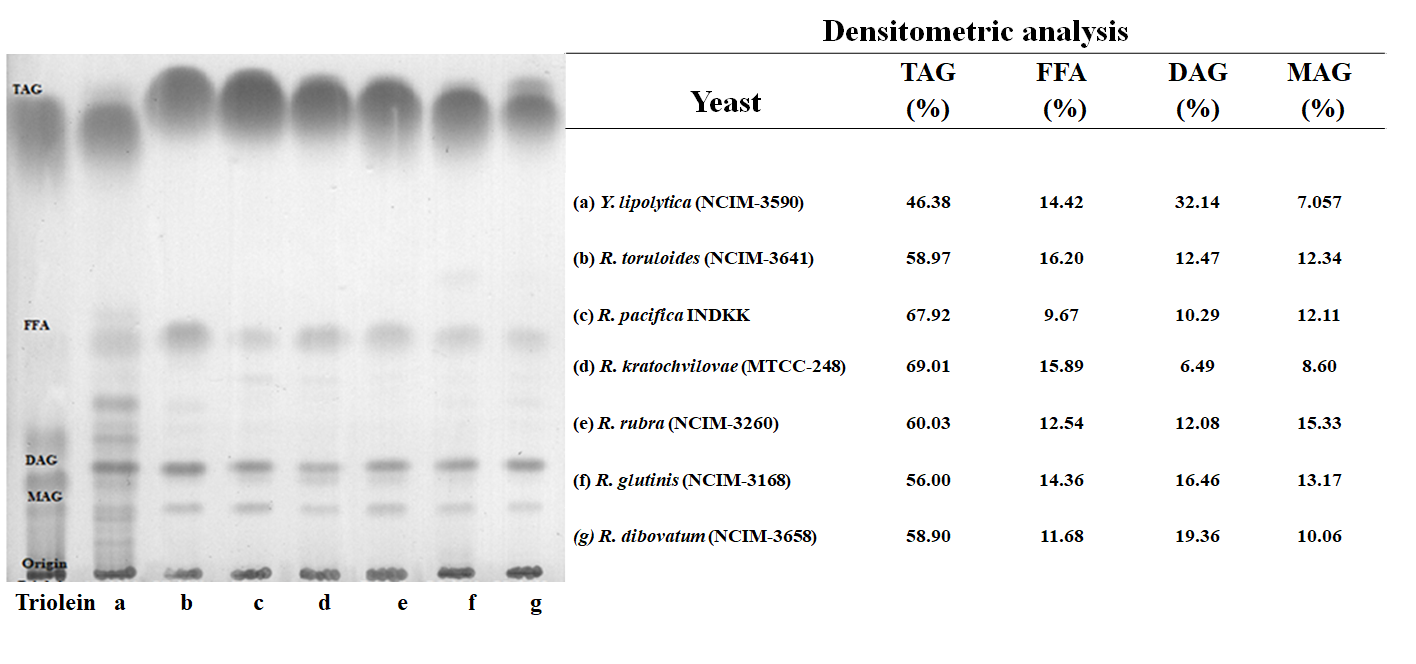
**
