# Supplementary material for: Harnessing pongamia shell hydrolysate for triacylglycerol agglomeration by novel oleaginous yeast Rhodotorula pacifica INDKK
Source: Biotechnol Biofuels. 2020 Oct 19;13:175. doi: 10.1186/s13068-020-01814-9 (PMC7574204; doi:10.1186/s13068-020-01814-9)
Supplement: Supplementary file 5 — Additional file 5: Table S2. Optimisation of Pre-treatment and saccharification process for pongamia shell hydrolysate (PSH) preparation. [file 13068_2020_1814_MOESM5_ESM.doc]

**Additional file -5**

**Table S2.** Optimisation of Pre-treatment and saccharification process for pongamia shell hydrolysate (PSH) preparation

| **Time** | **Treat- ment** | **Glucose**  **(g/L)** | **Xylose**  **(g/L)** | **Arabinose**  **(g/L)** | **Total sugars**  **(g/L)** | **HMF**  **(g/L)** | **Furfural**  **(g/L)** | **Acetic acid**  **(g/L)** |
| --- | --- | --- | --- | --- | --- | --- | --- | --- |
| 60  min | 0.5% H_2_SO_4_ | 1.43 | 0 | 1.71 | 3.14 | 0 | 0.056 | 0.68 |
|  | 1% H_2_SO_4_ | 1.89 | 1.89 | 3.82 | 9.53 | 0.012 | 0.056 | 2.15 |
|  | 1.5% H_2_SO_4_ | 2.17 | 7 | 2.23 | 11.40 | 0.012 | 0.037 | 3.44 |
|  | 2% H_2_SO_4_ | 2.22 | 21.52 | 5 | 28.74 | 0.041 | 0.039 | 6.04 |
| 90 min | 2% H_2_SO_4_ | 1.54 | 32.34 | 3.5 | 37.38 | 0.07 | 0.81 | 5.61 |
|  | 0.5% NaOH | 0 | 0.01 | 0 | 0.01 | 0.003 | 0 | 0.71 |
|  | 2% NaOH | 0 | 0.7 | 0 | 0.7 | 0.01 | 0 | 0.52 |
|  | | | | | | | | |
| **Time** | **Biomass load** | **Glucose**  **(g/L)** | **Xylose**  **(g/L)** | **Arabinose**  **(g/L)** | **Total sugars**  **(g/L)** | **HMF**  **(g/L)** | **Furfural**  **(g/L)** | **Acetic acid**  **(g/L)** |
|  | Saccharification at 50°C, 150 rpm, 20 FPU Enzyme (2% H_2_SO_4_ treated biomass) | | | | | | | |
| 72 h | 20% | 5.31 | 2.25 | 0.07 | 7.63 | 0 | 0.19 | 0.30 |
|  | Saccharification at 50°C, 150 rpm, 20 FPU Enzyme (2% NaOH treated biomass) | | | | | | | |
|  | 5% | 5.56 | 3.97 | 0.04 | 9.57 | 0.01 | 0 | 0.08 |
|  | 10% | 17.39 | 10.65 | 0 | 29.95 | 0.003 | 0 | 0.34 |
|  | 20% | 28.05 | 18.13 | 0.29 | 46.47 | 0.013 | 0 | 0.046 |
